# Non-Invasive Sex Determination of Nestlings and Adult Bonelli’s Eagles Using Morphometrics

**DOI:** 10.3390/ani13071201

**Published:** 2023-03-30

**Authors:** Irene Estellés-Domingo, Pascual López-López

**Affiliations:** Movement Ecology Lab, Cavanilles Institute of Biodiversity and Evolutionary Biology, University of Valencia, C/Catedrático José Beltrán 2, E-46980 Paterna, Valencia, Spain

**Keywords:** Accipitridae, birds, biometry, LDA, molecular sexing, PCR, raptors, reverse sexual dimorphism, sexing

## Abstract

**Simple Summary:**

This study explores the use of biometric analysis to identify the sex of Bonelli’s eagles, a bird species with reverse sexual dimorphism where females are usually larger than males. By using linear discriminant analysis of biometric variables, we were able to obtain equations that accurately distinguished between male and female eagles, using just two measurements. The study included 137 Bonelli’s eagles, 82 nestlings and 55 adults, sampled in eastern Spain from 2015 to 2022. The validation procedure reduced the number of variables used, increasing the accuracy of sexing and saving time. Results showed that the lateral tarsus length and dorso-ventral tarsus length measurements were the most effective in distinguishing between male and female eagles of all age classes, while other variables showed some overlap between sexes. This non-invasive method has multiple applications, including estimation of sex ratio for further studies on population dynamics and extinction risk assessments, which could contribute to the conservation of this endangered species.

**Abstract:**

Biometric analysis allows the sexing of most vertebrates, particularly birds. Birds of prey, and, especially, the Bonelli’s eagle (*Aquila fasciata*), show reverse sexual dimorphism (i.e., females are usually larger than males). In contrast to blood sampling, the use of morphometrics allows sex determination using a non-invasive method, and, therefore, it facilitates fieldwork. By means of a linear discriminant analysis of biometric variables, we obtained different equations that allow the sexing of nestlings and adult Bonelli’s eagles. We sampled 137 Bonelli’s eagles, 82 nestlings and 55 adults in eastern Spain during the period 2015–2022. The sexes obtained after linear discriminant analysis were compared with their molecular sexing. The validation procedure of the linear discriminant equations facilitated the reduction of the number of variables used and, consequently, optimised working time and sexing accuracy. After validation, some equations showed a 100% sexing efficiency for Bonelli’s eagles, particularly for adults. Our results showed that the variables with smaller overlap between the sexes were the lateral tarsus length and dorso-ventral tarsus length, particularly in nestlings. The rest of the variables showed some overlap between the sexes in both age classes. The results we obtained enable the sexing of juvenile and adult Bonelli’s eagles in the field using just these two measurements. Hence, this is an easy, accurate, quick and non-invasive method with multiple applications, including in studies on population dynamics, survival analysis or extinction risk assessments, which, ultimately, could contribute to the improvement of the conservation status of this endangered species.

## 1. Introduction

Sex determination is a procedure with multiple applications in, for example, ecology, including gene dispersal studies [1,2], survival studies [3], ethological studies [4] and conservation [5]. In addition, sex determination is key for conservation programs of endangered species and for species reintroduction projects [6,7]. Although most raptors have reversed sexual dimorphism, and females are usually larger than males [8,9,10], in some species, sex determination is particularly difficult as the raptors can apparently be monomorphic [11], showing an overlap in their size range [12,13].

Different techniques have been used for sexing raptors, including methods based on the study of phenotypic characteristics such as feathers and colours [14], morphometry (e.g., [13,15,16]), molecular techniques (e.g., [17,18,19]) or the combination of them (e.g., [20,21]). Morphometric techniques are commonly used in the field in combination with molecular methods. However, although DNA analysis can provide accurate sex identification, morphometry is less invasive and easier to apply in the field.

The Bonelli’s eagle (*Aquila fasciata*) is a cliff-nesting raptor that occurs throughout the mountains of the Palearctic, Afrotropical and Indomalayan regions [22,23]. Due to a large population decline of this species at the end of the 20th century, it has been listed as Vulnerable in Spain (Spanish Royal Decree 139/2011, which established the National Catalogue of Endangered Species) and listed as a Regionally Endangered species in several regions in Spain. 

Previous studies compared morphometric versus DNA techniques for sexing Bonelli’s eagle nestlings [24,25,26]. Other studies used morphometrics and plumage colour patterns to sex adults [14]. However, to the best of our knowledge, there has been no single study in which the sex of Bonelli’s eagle adults and nestlings of the same population has been determined using the same methods. 

In view of existing precedents, and, given the lack of agreement on which criterion is the most appropriate for sexing both adult and juvenile Bonelli’s eagles, this paper aims to develop simple equations to unify the sexing criteria for both age groups of the Bonelli’s eagle. In addition, in the case of the nestlings, the aim was to enable their sexing considering their age throughout their ontogenetic development in the nest, which has been overlooked in most studies. The application of a quick and non-invasive method could facilitate work on the conservation of the Bonelli’s eagle, in which the correct estimation of the sex ratio is an important factor in terms of the analysis of survival, population dynamics or the risk of extinction, and in other potential applications.

## 2. Material and Methods

### 2.1. Study Area

The study area is located in eastern Spain, including Albacete, Alicante, Castellón, Cuenca, and Valencia provinces. The area covers approximately 7600 km^2^, with altitudes ranging between the coastline and 1814 m above sea level. The climate is Mediterranean, with an average annual temperature that varies between 17 °C in the coastal areas and 8 °C in the mountains. The population size in the study area has decreased in the last decades, and, currently, less than one hundred pairs remain [27].

### 2.2. Sample Collection

We trapped adults in the framework of an ongoing GPS tracking project aimed at studying mortality risks and eagles’ ranging behaviour in eastern Spain (more details in [28,29,30,31]). Adults were trapped from June to December during 2015–2022. We installed a trap in each territory consisting of a folding ground net activated by remote control in the territories occupied by the pairs of interest throughout the study area. The trap was always under surveillance by the researchers who were hidden nearby. The trap was only activated once the target individual/s were inside. In most cases, both pair members of each territory, male and female, were trapped at the same time. Once trapped, adults were measured, weighted and fitted with a GPS/GSM datalogger in a backpack configuration. This procedure took a maximum time of 30 to 40 min (more details in [28,29,30]).

In order to obtain information on the biometry of juveniles (i.e., nestlings), field visits every two weeks were made during December, January and early February to determine the locations of the nests and to select which nests would be accessed [32]. During this period, we used 10 × 42 binoculars (Olympus, Tokyo, Japan), a Swarovski 20–60× telescope (Swarovski Optik, Absam, Austria) and a Nikon camera with a 55–300 mm lens (Nikon, Tokyo, Japan). We estimated the age of nestlings whose parents did not have GPS transmitters using the feather development patterns, following the methodology described in [33] and used in similar studies of the same species [32,34]. For those juveniles whose parents had GPS transmitters, we knew the exact laying date, and, consequently, their age was precisely assessed [28]. As for the adults, morphometric measurements were taken from juveniles after reaching the nest on the cliffs with the participation of expert climbers. We also took blood samples from all individuals in this study with a 25 G needle and a 5 mL syringe which were preserved in 3 mL EDTA and heparin tubes for further analyses. We also took oral swabs to check for the occurrence of *Trichomonas gallinae*, a protozoan parasite that causes juvenile mortality in the nest [35].

### 2.3. Molecular Sexing

All individuals were sexed by using molecular methods based on polymerase chain reaction (PCR) with PM and PN/p2 25 nmol primers (ThermoFisher Scientific, Waltham, MA, USA, product code 10336022) and separating the PCR product with a polyacrylamide gel. We followed the procedure described in [36]. 

### 2.4. Morphometric Measurements

All measurements of adults and nestlings were taken by the same person (Pascual López-López) to minimise measurement errors. All the measurements were taken with a metal ruler, calipers and a tape measure to the nearest 0.01 mm (Table 1). Body mass was measured with a digital balance to the nearest 1 g. 

### 2.5. Statistical Analyses

Descriptive statistical analyses were performed for each variable, including the mean, median, maximum, minimum and standard deviation. Data were divided into two age groups: juveniles and adult birds. The normality of the variables was checked by Shapiro–Wilk normality tests [37]. A nonparametric Mann–Whitney test [37] was then performed to test for differences between sexes for each of the variables. The effect size was calculated as the z-statistic divided by the square root of the sample size (*n*) using the “rstatix” R package [38]. The effect size value varied from 0 to close to 1 and was interpreted as follows: 0.10–<0.3 (small effect), 0.30–<0.5 (moderate effect) and ≥0.5 (large effect). We also computed the percentage of sexual dimorphism as 100 × [(male mean/female mean) − 1] for each variable [13]. Negative values indicated that the variable is larger in females than in males.

Secondly, all variables were standardised and then included in a linear discriminant analysis (LDA) to assess which combination of variables discriminates between males and females, generating the discriminant function [14,21,39]. The model was validated using a leave-one-out (jack-knife) procedure [40]. To check the effectiveness of the discriminant function, the results were compared with the results of the molecular sexing. 

Then, the morphometric measurements were eliminated from each equation one by one to determine which combination of variables best explains the differences between the sexes using Wilk’s lambda statistic [41]. In this way, it was possible to calculate the predictive capacity of the functions obtained using 2 × 2 contingency tables with their respective 95% confidence interval (CI). All the analyses and figures were prepared using R version 4.1.2 and the RStudio program environment [42] with the following packages: tydiverse [43], caret [44], dplyr [43], psycho [45], tibble [46] and corrplot [47]. The significance threshold was set at *p* < 0.05.

## 3. Results

Overall, we sampled 137 Bonelli’s eagles: 82 nestlings and 55 adults. Of these, we recorded complete information on all variables of 68 nestlings and 33 adults. According to the molecular analysis, the eagles’ sexes were as follows: 34 males and 34 females among nestlings and 17 males and 16 females among adults.

### 3.1. Bonelli’s Eagle Nestlings 

**Figure 1:**
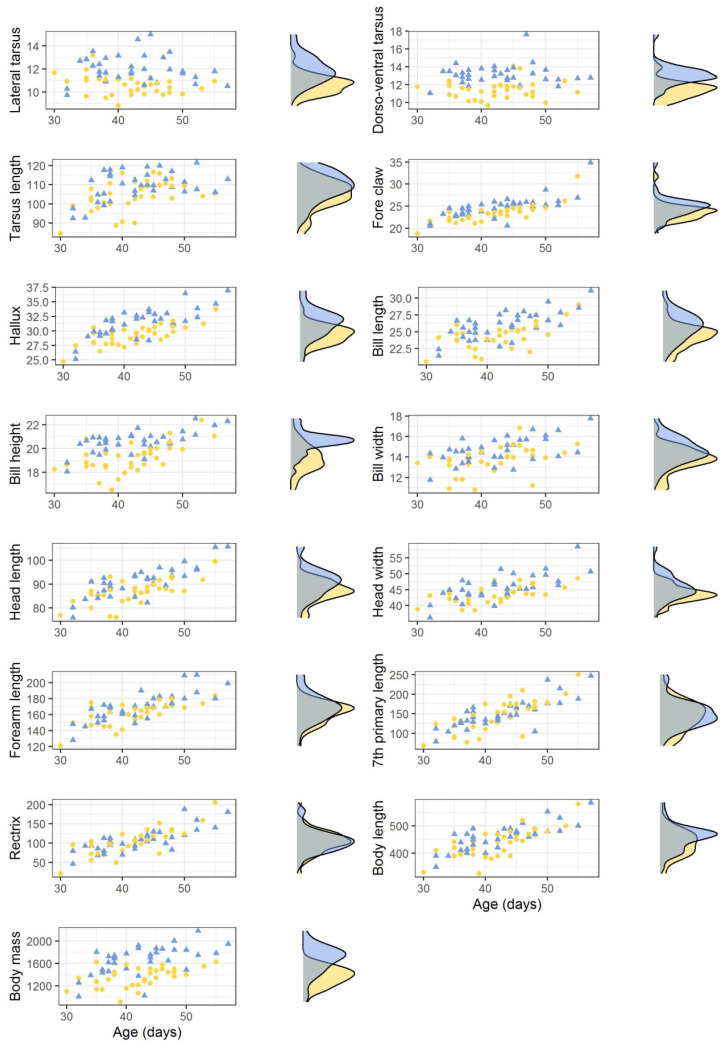
Morphometrics of male (yellow dots, *n* = 34) and female (blue triangles, *n* = 34) Bonelli’s eagle nestlings from eastern Spain sexed by molecular techniques. Measurements are given in mm and nestlings’ age in days. Density plots are shown to the right of each figure.

Morphometrics differed between the sexes (Table 2). In general, females of similar age were larger than males for all the variables described. However, there was some degree of overlap in all the variables (Figure 1).

The variables that showed the greatest dimorphism were the body mass (% dimorphism = −18.27%), the lateral tarsus (% dimorphism = −11.34%) and the dorso-ventral tarsus (% dimorphism = −12.87%), whereas those with the smallest were the rectrix feather (% dimorphism = −1.73%) and the seventh primary feather (% dimorphism = −3.77%). All variables showed significant differences between sex, excepting the rectrix and the seventh primary feather (Table 2).

Considering all variables, we obtained a linear classification equation for which the leave-one-out cross-validation showed an average of 84.54% accuracy in males (95%CI = 81.82–87.88%) and 87.62% in females (95%CI = 79.41–90.91%) (Equation (1)). In the LDA equations scores, >0 indicated females and <0 males. The discriminant function for Bonelli’s eagle nestlings considering all measured variables was as follows:

Sex score = 0.328 × (lateral tarsus) + 0.107 × (dorso-ventral tarsus) − 0.026 × (tarsus length) − 0.323 × (fore claw) + 0.644 × (hallux) − 0.097 × (bill length) + 0.706 × (bill height) + 0.383 × (bill width) − 0.003 × (head length) − 0.057 × (head width) − 0.011 × (forearm length) + 0.008 × (7th primary) − 0.036 × (rectrix) + 0.004 × (body length)(1)

Due to the large number of variables and the low statistical significance of the differences in the seventh primary feather, rectrix, tarsus length and forearm length, we decided to make an LDA with the easiest variables to measure (Equation (2)).

Sex score = 0.439 × (lateral tarsus) + 0.260 × (dorso-ventral tarsus) − 0.304 ×  (fore claw) + 0.443 × (hallux) − 0.093 × (bill length) + 0.662 × (bill height) + 0.378 × (bill width) + 0.016 × (head length) − 0.082 × (head width) − 0.011 ×  (body length)(2)

The cross-validated performance showed an average of 85.11% accuracy for sexing in males (95%CI = 81.82–90.91%) and 86.91% in females (95%CI = 81.82–90.91%) using Equation (2).

### 3.2. Bonelli’s Eagle Adults

**Figure 2:**
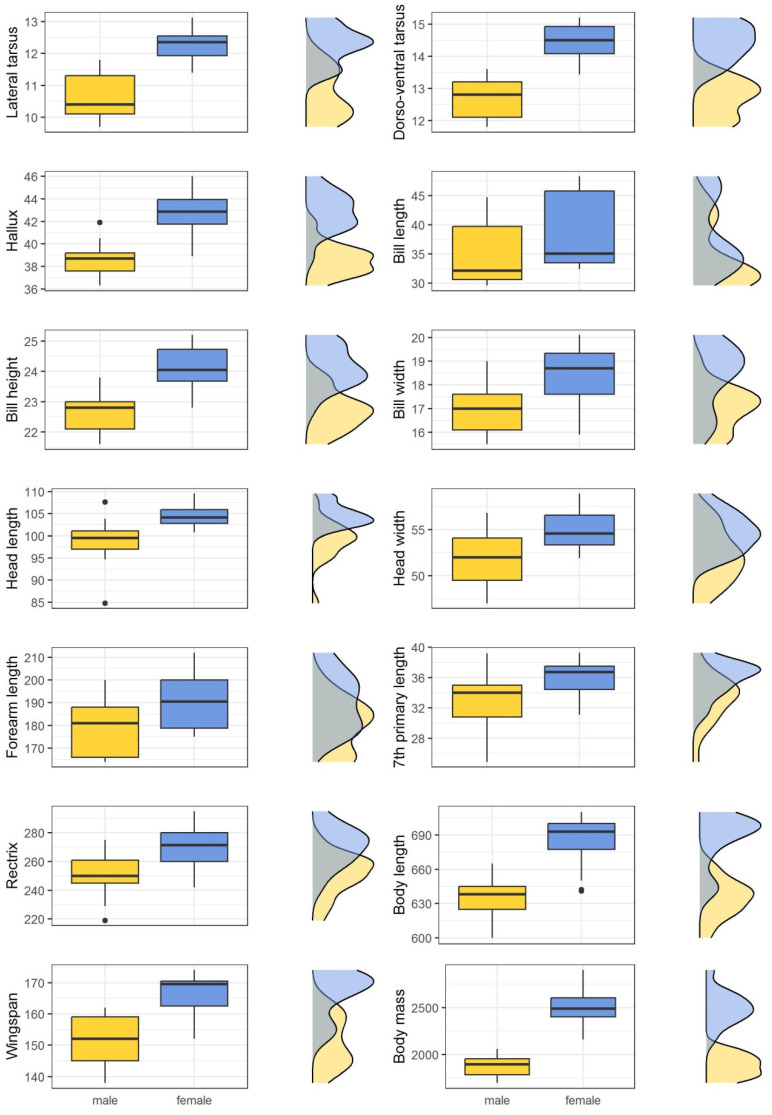
Morphometrics of adult male (*n* = 17) and female (*n* = 16) Bonelli’s eagles from eastern Spain sexed by molecular techniques. Measurements are given in mm. Density plots are shown to the right of each figure.

As in the nestlings, morphometrics differed between sexes, and females were larger than males in all the variables described (Table 3). There was overlap in all the variables but to a smaller extent than in the nestlings (Figure 2).

The greatest degree of sexual dimorphism was observed in body mass (% dimorphism = −24.65), lateral tarsus (% dimorphism = −12.70) and dorso-ventral tarsus (% dimorphism = −12.17), and the lowest degree of sexual dimorphism was obtained for head length (% dimorphism = −5.54%) and head width (% dimorphism = −4.95) (Table 3).

Considering all variables, we obtained a linear classification equation for adults for which the leave-one-out cross-validation showed an average of 94.30% accuracy in males (95%CI = 93.75–100%) and 93.75% in females (95%CI = 93.33–100%) (Equation (3)). As for nestlings, in this equation, scores >0 indicated females and <0 males. 

The discriminant function for Bonelli’s eagle adults considering all measured variables is as follows:

Sex score = 1.491 × (lateral tarsus) + 0.645 × (dorso-ventral tarsus)) + 0.453 × (hallux) + 0.186 × (bill length) + 0.380 × (bill height) − 0.450 × (bill width) − 0.119 × (head length) − 0.102 × (head width) + 0.044 × (forearm length) − 0.064 × (7th primary) + 0.026 × (rectrix) + 0.030 × (body length) − 0.008 × (wingspan)(3)

After the removal of the feather variables, the cross-validated performance of the LDA showed an average of 94.86% accuracy of sexing in males (95%CI = 93.75–100%) and 93.75% in females (95%CI = 93.33–100%) (Equation (4)).

Sex score = 1.452 × (lateral tarsus) + 0.647 × (dorso-ventral tarsus)) + 0.493 × (hallux) + 0.067 × (bill length) + 0.537 × (bill height) − 0.450 × (bill width) − 0.096 × (head length) − 0.072 × (head width) + 0.037 × (body length) − 0.017 × (wingspan)(4)

Due to the high significance of the dorso-ventral tarsus and the lateral tarsus, we built a model using just those measures. This model showed 100% accuracy for sexing in females (95%CI = 100–100%) and 93.54% in males (95%CI = 87.50–100%) (Equation (5)).

Sex score = 0.745 × (lateral tarsus) + 1.317 × (dorso-ventral tarsus)(5)

## 4. Discussion

Our results reaffirm that both nestling and adult Bonelli’s eagles show reverse sexual dimorphism, which is in agreement with previous works [14,25,26]. However, as reported in other raptors [48], adults and nestlings require different equations for sex discrimination as they show morphological differences. This happens because late-state nestlings of raptor species are known to have not yet fully developed, particularly in terms of their bills [49,50], hallux claws [51] and feathers [21,52].

We found that the lateral tarsus and the dorso-ventral tarsus showed the lowest variation throughout the development of the nestlings. Despite the allometric growth that characterises nestlings of birds of prey, both variables stabilise at early ages and, thus, allow the correct distinction between sexes.

The least statistically significant variables in the LDA for the nestlings were the lengths of the rectrix and of the seventh primary. This may be due to the fact that feathers in raptors generally vary only slightly in size between sexes [53,54], which was previously observed in Bonelli’s eagle nestlings [25], and because both variables showed high individual variation regardless of sex. 

In addition to the general function in which all variables were included, we developed a discriminant function in which only those variables that were easy to measure were taken into account (i.e., those excluding the feathers). The classification efficiency of more than 84% may be a consequence of the removal of the length of the feathers as sex predictors and the fact that the variables that were easy to measure were also variables that significantly contributed to the power of the discriminant functions. 

In contrast to previous studies in which body mass and feather length (wing and tail length) were selected as the best discriminant variables [26], our study shows that sex discrimination of nestlings can be easily obtained using variables with little variation throughout chicks’ ontogeny. This is particularly important in the case of variables such as body mass and feather length. In raptors, nestlings’ body mass and feather length show large variation between individuals [55,56]. The body mass varies in relation to food availability and even within individuals during the day [57,58]. Therefore, the use of these variables that do not stabilise their growth during chicks’ development is not recommended for sex determination.

In the case of adults, we confirmed previous studies in which Bonelli’s eagles showed a marked reverse sexual dimorphism [14], being one of the Palaearctic species with the greatest size differences between sexes [23]. In contrast to García et al. [14], which did not find any overlap in body length and hallux length between sexes and, thus, recommended these variables for sex determination, we found a slight degree of overlap among individuals (Figure 2). This limits their application as single variables for sex determination.

The adults’ equation including all the variables showed a 94% average success rate in sexing. This function may not have been fully effective due to the inclusion of many variables with high overlap. Furthermore, the equation without feather variables showed an average 95% success rate in males and 94% in females. Interestingly, the equation with just the lateral tarsus and the dorso-ventral tarsus measurements included as predictors showed 100% accuracy in females and 94% in males. 

Despite the high percentage of sexual dimorphism, the body mass showed high individual variation and some degree of overlap among individuals (Figure 2). In fact, we weighted the same individuals at different times because we trapped some of them several times because the GPS transmitter had fallen off. We found differences in the body mass of these individuals throughout the annual cycle. In agreement with García et al. [14], the weight of individuals can vary up to 10% depending on physical condition, the ecological characteristics of the foraging area and the phase of the year (i.e., the highest body mass values were recorded in months prior to reproduction). Therefore, body mass should be used with caution in sex determination in the field.

Finally, previous studies suggested that the differences in size observed across Bonelli’s eagle subpopulations could reflect biological differences among them [14,26]. However, we consider that these small differences might be better explained by the fact that measurements were taken by different researchers, at least in [26], which could have affected the result. In our opinion, the changes in individual size can be explained solely by the different ways of measuring used by different researchers or by limited sample size. The metapopulation structure of the Bonelli’s eagle [59,60], in which some exchange of individuals between the Iberian and southern French regions occurs [1,2,61,62], does not favour any actual sub-regional differences.

## 5. Conclusions

Our results show that simple equations provide a great advantage for rapid sex determination in the field with small error. This is particularly important for nestlings, for which nests must be accessed by experienced climbers during a short period of development, usually when chicks are 35–45 days old. Taking body measurements when chicks are younger than 25–30 days old might result in nest abandonment or the nestlings’ death as a result of their lower thermoregulation ability. On the other hand, manipulation of chicks older than 50–55 days might result in nestlings’ premature abandonment of the nest as the climbers approach, causing serious injuries or even death in extreme cases. Thus, the availability of simple methods for quick sex determination is essential when the manipulation time is limited. 

In conclusion, we consider the equations we proposed to allow the quick sexing of juvenile and adult Bonelli’s eagles in the field is an easy, accurate and non-invasive approach with multiple applications. This is particularly useful for studies on population dynamics and survival analysis or extinction risk assessments that, ultimately, could contribute to the improvement of the conservation status of this endangered species.

## Figures and Tables

**Table 1:** Measurements taken from adult and juvenile Bonelli’s eagles in eastern Spain in 2015–2022.

Variable	Description	Instrument	Units
Lateral tarsus	Lateral width of tarsus-metatarsus in the narrowing part situated above the beginning of the metatarsal angle.	Calipers	mm
Dorso-ventral tarsus	Dorso-ventral width of tarsus-metatarsus in the narrowing part situated above the beginning of the metatarsal angle.	Calipers	mm
Tarsus length	Distance from the tarsometatarsal joint to the articulation of the middle toe.	Calipers	mm
Fore claw length	Length of the claw of the central toe.	Calipers	mm
Hallux length	Length of the claw of the hind toe.	Calipers	mm
Bill length	Length from the distal end of the bill to the distal dorsal edge of the cere.	Calipers	mm
Bill height	Length from the distal edge of the cere to the base of the bill, placing the caliper perpendicular to the bill.	Calipers	mm
Bill width	Width of the bill taken by the calipers placed against the corners of the bill at the level of the nostrils.	Calipers	mm
Head width	Width of the skull measured behind the eyes in the auricular region.	Calipers	mm
Head length	Maximum length from the back of the skull to the distal end of the bill.	Calipers	mm
Seventh primary length	Length of the seventh primary feather (counting from the outer tip of the wing inwards) to its insertion.	Metal ruler	mm
Forearm length	Length of the forearm ventral to the shoulder, positioning the ruler at the elbow to the distal end of the ulna.	Metal ruler	mm
Rectrix	Length of the central feather of the tail to its insertion.	Metal ruler	mm
Wingspan	Distance between the tips of the extended wings.	Tape measure	mm
Body length	Length from the end of the bill to the end of the central feather of the tail with the bird stretched out on a table.	Tape measure	mm
Body mass	Body mass weighted on a digital balance.	Digital balance	g

**Table 2:** Summary statistics and results of the Mann–Whitney test of morphometric measurements of male (*n* = 34) and female (*n* = 34) Bonelli’s eagles nestlings from eastern Spain sexed by molecular techniques. Values are given in mm except body mass which is given in grams. The importance of each variable in the identification of the sexes with respect to the other variables (effect size) and the percentage of sexual dimorphism are shown.

Variable	Sex	Mean	sd	Median	Min	Max	*p*-Value	Effect Size	% Dimorphism
Lateral tarsus	male	10.60	0.83	10.65	8.81	13.16	<0.001	0.577	−11.34
	female	11.96	1.18	11.77	9.72	14.98			
Dorso-ventral tarsus	male	11.40	0.96	11.43	9.64	14.08	<0.001	0.676	−12.87
	female	13.09	1.15	12.98	11.03	17.62			
Tarsus length	male	104.43	7.92	104.71	84.53	116.55	0.006	0.309	−4.55
	female	109.41	7.44	109.76	92.46	121.55			
Fore claw	male	23.48	2.08	23.64	18.78	31.74	0.002	0.344	−5.19
	female	24.77	2.54	24.96	20.47	34.88			
Hallux	male	29.27	1.72	29.52	24.70	33.73	<0.001	0.476	−6.77
	female	31.40	2.48	31.67	25.12	36.96			
Bill length	male	24.32	1.79	24.50	20.58	28.99	0.001	0.397	−6.21
	female	25.93	2.08	25.96	21.42	31.12			
Bill height	male	19.23	1.22	19.30	16.53	22.40	<0.001	0.543	−6.53
	female	20.58	0.93	20.68	18.06	22.53			
Bill width	male	13.68	1.32	13.86	10.79	16.84	0.002	0.356	−6.73
	female	14.66	1.26	14.55	11.75	17.76			
Head length	male	87.02	5.04	87.15	76.14	99.47	0.003	0.335	−4.24
	female	90.87	6.38	90.76	75.93	105.71			
Head width	male	43.48	2.56	43.46	38.42	48.54	0.008	0.295	−4.72
	female	45.64	4.16	45.23	36.14	58.54			
Forearm length	male	161.41	13.92	165.50	121.00	184.00	0.032	0.225	−4.80
	female	169.56	17.57	170.00	128.00	210.00			
Seventh primary	male	145.47	41.06	149.50	68.00	250.00	0.352	0.047	−3.77
	female	151.18	36.22	146.50	79.00	247.00			
Rectrix	male	105.41	33.99	105.00	21.00	205.00	0.527	0.001	−1.73
	female	107.27	30.13	104.50	46.00	188.00			
Body length	male	436.87	52.08	440.00	325.00	580.00	0.024	0.240	−5.03
	female	460.03	47.68	465.00	349.00	585.00			
Body mass	male	1359.0	171.0	1387.50	920	1625	<0.001	0.597	−18.27
	female	1662.9	254.1	1725.00	1015	2180			

**Table 3:** Summary statistics and results of the Mann–Whitney test of morphometric measurements of adult male (*n* = 17) and female (*n* = 16) Bonelli’s eagles from eastern Spain sexed by molecular techniques. Values are given in mm except body mass which is given in grams. The importance of each variable in the identification of the sexes with respect to the other variables (effect size) and the percentage of sexual dimorphism are shown.

Variable	Sex	Mean	sd	Median	Min	Max	*p*-Value	Effect Size	% Dimorphism
Lateral tarsus	male	10.70	0.75	10.40	9.70	11.80	<0.001	0.769	−12.70
	female	12.26	0.57	12.35	11.40	13.12			
Dorso-ventral tarsus	male	12.68	0.58	12.80	11.80	13.60	<0.001	0.844	−12.17
	female	14.44	0.57	14.50	13.44	15.20			
Hallux	male	38.59	1.39	38.70	36.30	41.90	<0.001	0.771	−9.90
	female	42.82	1.79	42.85	38.90	46.00			
Bill length	male	34.41	5.21	32.12	29.60	44.70	0.002	0.514	−10.97
	female	38.65	6.29	35.05	32.45	48.30			
Bill height	male	22.69	0.63	22.80	21.60	23.80	<0.001	0.738	−6.01
	female	24.15	0.68	24.05	22.80	25.20			
Bill width	male	16.97	0.96	17.00	15.50	19.00	0.002	0.505	−7.68
	female	18.38	1.36	18.70	15.90	20.10			
Head length	male	98.93	4.89	99.50	84.80	107.60	<0.001	0.677	−5.54
	female	104.72	2.51	104.15	100.80	109.50			
Head width	male	52.07	2.90	52.00	47.00	56.80	0.004	0.464	−4.95
	female	54.79	2.13	54.55	51.90	58.90			
Forearm length	male	179.47	11.34	181.00	164.00	200.00	0.017	0.373	−5.94
	female	190.81	12.37	190.50	175.00	212.00			
Seventh primary	male	33.15	3.34	34.00	24.90	39.20	0.003	0.484	−7.64
	female	35.89	2.23	36.75	31.10	39.30			
Rectrix	male	250.53	14.37	250.00	219.00	275.00	<0.001	0.587	−7.43
	female	270.63	13.73	271.50	242.00	295.00			
Body length	male	634.94	17.06	638.00	600.00	665.00	<0.001	0.773	−7.38
	female	685.56	22.75	693.00	641.00	710.00			
Wingspan	male	151.18	7.88	152.00	138.00	162.00	<0.001	0.694	−9.03
	female	166.19	7.34	169.50	152.00	174.00			
Body mass	male	1882.2	115.2	1894.00	1700	2060	<0.001	0.851	−24.65
	female	2498.1	182.9	2490.00	2160	2900			

## Data Availability

All data used in this study are available upon request to authors.

## References

[1] Cadahía L., López-López P., Urios V., Soutullo Á., Negro J.J. (2009). Natal dispersal and recruitment of two Bonelli’s Eagles Aquila fasciata: A four-year satellite tracking study. Acta Ornithol..

[2] Cadahía L., López-López P., Urios V., Negro J.J. (2010). Satellite telemetry reveals individual variation in juvenile Bonelli’s eagle dispersal areas. Eur. J. Wildl. Res..

[3] Newton I., Marquiss M., Rothery P. (1983). Age structure and survival in a sparrowhawk population. J. Anim. Ecol..

[4] Clutton-Brock T. (1986). Sex ratio variation in birds. Ibis.

[5] Griffiths R., Tiwari B. (1995). Sex of the last wild Spix’s macaw. Nature.

[6] Muriel R., Casado E., Schmidt D., Calabuig C.P., Ferrer M. (2010). Morphometric sex determination of young Ospreys Pandion haliaetus using discriminant analysis. Bird Study.

[7] Ferrer M., Delecourt C. (1992). Sex identification in the Spanish imperial eagle. J. Field Ornithol..

[8] Wheeler P., Greenwood P.J. (1983). The evolution of reversed sexual dimorphism in birds of prey. Oikos.

[9] Montgomerie R., Lundberg A. (1989). Reversed sexual dimorphism in raptors: Which sex changed size?. Oikos.

[10] Schoenjahn J., Pavey C.R., Walter G.H. (2020). Why female birds of prey are larger than males. Biol. J. Linn. Soc..

[11] Krüger O. (2005). The evolution of reversed sexual size dimorphism in hawks, falcons and owls: A comparative study. Evol. Ecol..

[12] Morrison J., Maltbie M. (1999). Methods for gender determination of Crested Caracaras. J. Raptor Res..

[13] López-López P., Gil J.A., Alcántara M. (2011). Morphometrics and sex determination in the endangered Bearded Vulture (*Gypaetus barbatus*). J. Raptor Res..

[14] García V., Moreno-Opo R., Tintó A. (2013). Sex differentiation of Bonelli’s eagle Aquila fasciata in western europe using morphometrics and plumage colour patterns. Ardeola.

[15] Harmata A., Montopoli G. (2013). Morphometric sex determination of North American Golden Eagles. J. Raptor Res..

[16] Boucheker A., Nedjah R., Prodon R., Gillingham M., Dechaume-Moncharmont F.-X., Béchet A., Samraoui B. (2020). Cohort effect on discriminant rate: The case of greater flamingo (*Phoenicopterus roseus*) chicks sexed with morphological characters. Web Ecol..

[17] Griffiths R., Daan S., Dijkstra C. (1996). Sex identification in birds using two CHD genes. Proc. R. Soc. London. Ser. B Biol. Sci..

[18] Fridolfsson A.-K., Ellegren H. (1999). A simple and universal method for molecular sexing of non-ratite birds. J. Avian Biol..

[19] Dubiec A., Zagalska-Neubauer M. (2006). Molecular techniques for sex identification in birds. Biol. Lett..

[20] Balbontín J., Ferrer M., Casado E. (2001). Sex determination in booted eagles (*Hieraaetus pennatus*) using molecular procedures and discrimiant function analysis. J. Raptor Res..

[21] Dykstra C.R., Mays H.L., Hays J.L., Simon M.M., Wegman A.R. (2012). Sexing Adult and Nestling Red-Shouldered Hawks Using Morphometrics and Molecular Techniques1. J. Raptor Res..

[22] Cramp S., Ferguson-Lees I.J., Gillmor R., Hollom P., Hudson R., Nicholson E.M., Ogilvie M., Olney P., Voous K., Wattel J. (1983). The Birds of the Western Palearctic.

[23] Ferguson-Lees J., Christie D.A. (2001). Raptors of the World.

[24] Mañosa S., Codina J. (1995). Age estimation and growth patterns in nestling Bonelli’s Eagles. J. Raptor Res..

[25] Palma L., Mira S., Cardia P., Beja P., Guillemaud T., Ferrand N., Cancela M., da Fonseca L. (2001). Sexing Bonelli’s Eagle nestlings: Morphometrics versus molecular techniques. J. Raptor Res..

[26] Redondo-Gómez D., Bautista J., Gil-Sánchez J., Pares F., Hernandez-Matias A., Resano-Mayor J., Real J., Pacteau C., Madero A., Moleón M. (2022). Towards accurate and simple morphometric sex differentiation in Bonelli’s Eagle Aquila fasciata nestlings: Interpopulation variations and influence of growth conditions. Avian Biol. Res..

[27] Del Moral J.C., Molina B. (2018). El águila Perdicera en España, Población Reproductora en 2018 y Método de Censo.

[28] López-López P., Perona A., Egea-Casas O., Morant J., Urios V. (2022). Tri-axial accelerometry shows differences in energy expenditure and parental effort throughout the breeding season in long-lived raptors. Curr. Zool..

[29] Morollón S., Urios V., López-López P. (2022). Home-Range Size and Space Use of Territorial Bonelli’s Eagles (*Aquila fasciata*) Tracked by High-Resolution GPS/GSM Telemetry. Diversity.

[30] Morollón S., Urios V., López-López P. (2022). Fifteen days are enough to estimate home-range size in some long-lived resident eagles. J. Ornithol..

[31] Perona A.M., Urios V., López-López P. (2019). Holidays? Not for all. Eagles have larger home ranges on holidays as a consequence of human disturbance. Biol. Conserv..

[32] López-López P., García-Ripollés C., Urios V. (2007). Population size, breeding performance and territory quality of Bonelli’s eagle Hieraaetus fasciatus in eastern Spain. Bird Study.

[33] Gil-Sánchez J.M. (2000). Efecto de la altitud y de la disponibilidad de presas en la fecha de puesta del águila-azor perdicera (*Hieraaetus fasciatus*) en la provincia de Granada (SE de España). Ardeola.

[34] López-López P. (2022). Potential negative effects of the installation of video surveillance cameras in raptors’ nests. Sci. Rep..

[35] Sansano-Maestre J., Garijo-Toledo M., Gomez-Munoz M. (2009). Prevalence and genotyping of Trichomonas gallinae in pigeons and birds of prey. Avian Pathol..

[36] Ito H., Sudo-Yamaji A., Abe M., Murase T., Tsubota T. (2003). Sex identification by alternative polymerase chain reaction methods in Falconiformes. Zool. Sci..

[37] Zar J. (2014). Biostatistical Analysis.

[38] Kassambara A. Rstatix: Pipe-Friendly Framework for Basic Statistical Tests. 2023. Version 0.7.2. https://CRAN.R-project.org/package=rstatix.

[39] Dillon W.R., Goldstein M. (1984). Multivariate Analysis: Methods and Applications.

[40] Dechaume-Moncharmont F.-X., Monceau K., Cezilly F. (2011). Sexing birds using discriminant function analysis: A critical appraisal. Auk.

[41] McLachlan G.J. (2005). Discriminant Analysis and Statistical Pattern Recognition.

[42] R Core Team R: A Language and Environment for Statistical Computing 2022. http://www.r-project.org/index.html.

[43] Wickham H., François R., Henry L., Müller K., Vaughan D. Posit PBC Dplyr: A Grammar of Data Manipulation 2023. Version 1.1.1. https://CRAN.R-project.org/package=dplyr.

[44] Kuhnaut M., Wing J., Weston S., Williams A., Keefer C., Engelhardt A., Cooper T., Mayer Z., Kenkel B., R Core Team (2022). Caret: Classification and Regression Training 2022.

[45] Makowskiaut D., Najberg H., Simko V., Epskamp S. psycho: Efficient and Publishing-Oriented Workflow for Psychological Science 2021. Version 0.6.1. https://CRAN.R-project.org/package=psycho.

[46] Müller K., Wickham H., Francois R., Bryan J. Tibble: Simple Data Frames 2022. Version 3.2.1. https://CRAN.R-project.org/package=tibble.

[47] Wei T., Simko V., Levy M., Xie Y., Jin Y., Zemla J., Freidank M., Cai J., Protivinsky T. Corrplot: Visualization of a Correlation Matrix 2021. Version 0.92. https://CRAN.R-project.org/package=corrplot.

[48] Pay J.M., Katzner T.E., Wiersma J.M., Brown W.E., Hawkins C.E., Proft K.M., Cameron E.Z. (2021). Morphometric Sex Identification of Nestling and Free-Flying Tasmanian Wedge-Tailed Eagles (*Aquila audax fleayi*). J. Raptor Res..

[49] Bortolotti G.R. (1984). Physical development of nestling bald eagles with emphasis on the timing of growth events. Wilson Bull..

[50] Donohue K.C., Dufty A.M. (2006). Sex determination of Red-tailed Hawks (*Buteo jamaicensis calurus*) using DNA analysis and morphometrics. J. Field Ornithol..

[51] Bortolotti G.R. (1986). Influence of sibling competition on nestling sex ratios of sexually dimorphic birds. Am. Nat..

[52] McPherson S.C., Brown M., Downs C.T. (2017). Gender-related morphometric differences in mature and nestling Crowned Eagles, with comments on ringing of eagle nestlings in KwaZulu-Natal, South Africa. Ostrich.

[53] Poole K. (1989). Determining age and sex of nestling Gyrfalcons. J. Raptor Res..

[54] Sodhi N. (1992). Growth of nestling Merlins, Falco columbarius. Can. Field-Nat. Ott. ON.

[55] Choi C.-Y., Nam H.-Y., Park J.-G., Bing G.-C., Park C., Cho S.-Y. (2013). Morphometrics and sexual dimorphism of Chinese Goshawks (*Accipiter soloensis*). J. Raptor Res..

[56] Smith N.R., Afton A.D., Hess T.J. (2016). Morphometric sex determination of after-hatch-year Bald Eagles in Louisiana. J. Raptor Res..

[57] Barton N.W., Houston D. (1993). A comparison of digestive efficiency in birds of prey. Ibis.

[58] Bordner Z.E., McCabe R.A., Brinker D., Rosenfield R.N., Jacobs E.A., England C., Wilson M., Goodrich L.J. (2022). Broad-Winged Hawk Size Varies by Sex and Latitude in North America. J. Raptor Res..

[59] Soutullo A., López-López P., Urios V. (2008). Incorporating spatial structure and stochasticity in endangered Bonelli’s eagle’s population models: Implications for conservation and management. Biol. Conserv..

[60] Hernandez-Matias A., Real J., Moleon M., Palma L., Sanchez-Zapata J., Pradel R., Carrete M., Gil-Sanchez J., Beja P., Balbontin J. (2013). From local monitoring to a broad-scale viability assessment: A case study for the Bonelli’s Eagle in western Europe. Ecol. Monogr..

[61] Real J., Manosa S. (2001). Dispersal of juvenile and immature Bonelli’s Eagles in northeastern Spain. J. Raptor Res..

[62] Hernandez-Matias A., Real J., Pradel R., Ravayrol A., Vincent-Martin N., Bosca F., Cheylan G. (2010). Determinants of territorial recruitment in Bonelli’s eagle (*Aquila fasciata*) populations. Auk.

